# Development and Validation of a Decision Analytical Model for Posttreatment Surveillance for Patients With Oropharyngeal Carcinoma

**DOI:** 10.1001/jamanetworkopen.2022.7240

**Published:** 2022-04-13

**Authors:** Vivek Nair, Samuel Auger, Sara Kochanny, Frederick M. Howard, Daniel Ginat, Olga Pasternak-Wise, Aditya Juloori, Matthew Koshy, Evgeny Izumchenko, Nishant Agrawal, Ari Rosenberg, Everett E. Vokes, M. Reza Skandari, Alexander T. Pearson

**Affiliations:** 1 University of Chicago Pritzker School of Medicine, Chicago, Illinois; 2 Department of Surgery, University of Chicago, Chicago, Illinois; 3 Department of Medicine, Section of Hematology/Oncology, University of Chicago, Chicago, Illinois; 4 Department of Radiology, University of Chicago, Chicago, Illinois; 5 Department of Radiation and Cellular Oncology, University of Chicago, Chicago, Illinois; 6 Centre for Health Economics and Policy Innovation, Imperial College Business School, Imperial College London, London, United Kingdom

## Abstract

**Importance:**

Clinical practice regarding posttreatment radiologic surveillance for patients with oropharyngeal carcinoma (OPC) is neither adapted to individual patient risk nor fully evidence based.

**Objectives:**

To construct a microsimulation model for posttreatment OPC progression and use it to optimize surveillance strategies while accounting for both tumor stage and human papillomavirus (HPV) status.

**Design, Setting, and Participants:**

In this decision analytical modeling study, a Markov model of 3-year posttreatment patient trajectories was created. The training data source was the American College of Surgeon’s National Cancer Database from 2010 to 2015. The external validation data set was the 2016 International Collaboration on Oropharyngeal Cancer Network for Staging (ICON-S) study. Training data comprised 2159 patients with OPC treated with primary radiotherapy who had known HPV status and disease staging information. Patients with American Joint Committee on Cancer, 7th edition stage III to IVB disease and those with clinical metastases during the time of primary treatment were included. Data were analyzed from August 1 to October 31, 2020.

**Main Outcomes and Measures:**

Main outcomes included disease stage and HPV status, specific disease transition probabilities, and latency of surveillance regimens, defined as time between recurrence incidence and disease discovery.

**Results:**

Training data consisted of 2159 total patients (1708 men [79.1%]; median age, 59.6 years [range, 40-90 years]; 401 with stage III disease, 1415 with stage IVA disease, and 343 with stage IVB disease). Cohorts predominantly had HPV-negative disease (1606 [74.4%]). With model-optimized regimens, recurrent disease was discovered a mean of 0.6 months (95% CI, 0.5-0.8 months) earlier than with a standard surveillance regimen based on current clinical guidelines. Recurrent disease was discovered using the optimized regimens without significant reduction in sensitivity. Compared with strategies based on reimbursement guidelines, the model-optimized regimens found disease a mean of 1.8 months (95% CI, 1.3-2.3 months) earlier.

**Conclusions and Relevance:**

Optimized, risk-stratified surveillance regimens consistently outperformed nonoptimized strategies. These gains were obtained without requiring any additional imaging studies. This approach to risk-stratified surveillance optimization is generalizable to a broad range of tumor types and risk factors.

## Key Points

**Question:**

Can risk-stratified posttreatment surveillance regimens for oropharyngeal carcinoma based on a decision analytical model outperform strategies based on current clinical practice?

**Findings:**

In this decision analytical modeling study, strategies optimized for tumor stage and human papillomavirus status were associated with a lower mean surveillance latency, defined as time between onset of recurrence of oropharyngeal carcinoma and its radiologic discovery, compared with common clinical guidelines. Compared with common medical reimbursement guidelines, model-optimized strategies were associated with lower detection latency without requiring any additional imaging studies.

**Meaning:**

This study suggests that radiologic surveillance strategies optimized using patient and tumor risk factors may result in earlier detection of recurrent oropharyngeal carcinoma compared with current paradigms.

## Teaser

This study uses decision analytical modeling to construct a microsimulation model for posttreatment oropharyngeal carcinoma progression and uses it to optimize surveillance strategies while accounting for both tumor stage and human papillomavirus status.

## Introduction

Recurrent head and neck cancer (HNC) is associated with poor outcomes, with most patients dying within 1 year of recurrence.^1,2^ Posttreatment radiologic surveillance is regularly used to monitor for recurrent disease and initiate early treatment. Most follow-up strategies have not been shown to improve patient survival.^3,4^ However, a posttreatment positron emission tomography (PET) scan can accurately detect recurrent disease after definitive radiotherapy when obtained at least 3 months after treatment.^5,6^ A randomized clinical trial has confirmed that negative, early PET scan results can obviate the need for neck dissection among patients with advanced nodal disease^7^; thus, the use of a single posttreatment PET scan is recommended in both clinical practice^8^ and reimbursement guidelines.^9^

Despite the lack of trial support,^8^ computed tomography (CT) scans of the neck and chest are also frequently used to monitor for recurrence of disease. Prior studies have demonstrated that additional imaging can increase the rate of detection of recurrences, although no survival benefit has been shown.^10^ Nonetheless, additional imaging surveillance is attractive because early recurrences are more amenable to salvage therapy.^11^ Because HNC surveillance guidelines vary significantly across institutions, there is a need for new, evidence-based tools to compare the effectiveness of different strategies.

One potential solution lies in mathematical models of cancer recurrence.^12^ Such methods have been widely used to optimize both oncologic^13,14^ and nononcologic^15,16^ interventions. Among the variety of approaches used by other groups, such as the nonlinear optimization techniques of Kent et al,^14^ one particularly attractive method is that of a Markov model. Markov models simulate the progression of multiple predefined states over time.^17^ They can capture the specific incidence and prognosis of local vs metastatic recurrence as well as false-positive and false-negative rates for different imaging studies.^18,19,20,21^ Markov models have already been used to simulate cancer recurrence.^22,23,24,25,26,27,28^ However, many models lack disease-specific risk stratification.

We focused on oropharyngeal carcinoma (OPC), a subset of HNC of increasing incidence.^29^ Oropharyngeal carcinomas can be divided by 2 key risk factors: stage and tumor human papillomavirus (HPV) status.^30^ These traits confer different rates of recurrence and patient survival.^31^ Human papillomavirus–associated tumor pathogenesis is thought to be due to a distinct mechanism of virally mediated mutagenesis. The demographic characteristics of patients with HPV-positive disease are also markedly different than those of patients with HPV-negative disease, with the former tending to be younger, of a higher socioeconomic status, and with a less significant smoking history.^32^ Patients with HPV-positive or earlier-stage disease thus tend toward better outcomes and may be best served by different surveillance schedules.^33^

Our goal was to construct a microsimulation model for OPC with tumor stage and HPV status risk stratification. We also sought to use our model to explore risk-optimized surveillance schedules for the first 3 years after treatment. We hypothesized that these model-designed regimens could outperform strategies based on current clinical and insurance guidelines.

## Methods

This decision analytical modeling study was reviewed and approved by the University of Chicago institutional review board. The American College of Surgeon’s National Cancer Database (NCDB) is a deidentified database in which participants provided written consent for inclusion. Data included in the NCDB have been stripped of direct identifiers to be compliant with the Health Insurance Portability and Accountability Act of 1996 as per 45 CFR § 164.514 (b); as such, individual consent was not required. This study was performed in accordance with the Standards for Reporting of Diagnostic Accuracy (STARD) reporting guideline for prognostic studies.^34^ All analysis was conducted from August 1 to October 31, 2020.

### Development of Pretraining Markov Models

**Figure 1.**
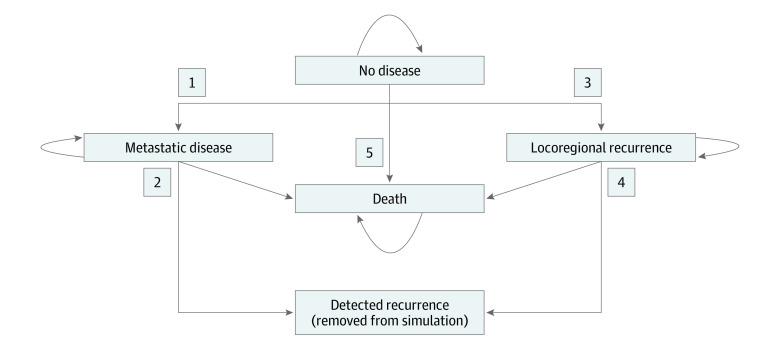
Markov Model Representation of Disease Progression Numbers adjacent to arrows correspond to sections in Table 1.

First, a disease progression model to simulate patient outcomes after cancer treatment was created (Figure 1). All patients were assumed to begin in a state of no disease. The transition between no disease and death was defined as death not related to tumor and was the same for the HPV-positive and HPV-negative cohorts.^35^ We included a functional state of detected recurrence, which refers to recurrence detected using radiologic surveillance. Any patient with detected recurrence was removed from the cohort. The training workflow and the data sets used at each step are shown in eFigure 1 in the Supplement.

The pretraining transition probabilities were derived from the literature available via PubMed/MEDLINE. Studies were found using Pubmed/MEDLINE searches with combinations of the terms *HPV*, *oropharyngeal carcinoma*, *local*, *distant*, *metastatic*, *recurrence*, *treatment failure*, and *disease progression*. Studies with the largest patient cohorts were selected. The search was conducted in September 2020. We selected 4 studies with extractable transition probabilities and HPV-stratified cohorts (Table 1).^18,19,20,35^

**Table 1.** Transition Probabilities and the Studies Used to Fit the Base Markov Model^a^

Disease state transition	Monthly transition probability	
	HPV positive, %	HPV negative, %
**1. No disease to distant metastases** ^20^		
Year		
1	0.3	0.8
2	0.3	0.6
≥3	0.2	0.1
**2. Distant metastases to death** ^20^		
Year		
1	10.2	19.4
2	8.1	11.8
≥3	5.6	11.8
**3. No disease to locoregional recurrence** ^18^		
Year		
1	0.2	2.7
2	0.1	1.6
≥3	0.1	0.8
**4. Locoregional recurrence to death** ^19^		
Year		
1	2.8	6.5
2	1.8	4.6
≥3	1.8	4.4
**5. No disease to death** ^35^		
Year		
1	0.4	0.4
2	0.2	0.2
≥3	0.1	0.1

Abbreviation: HPV, human papillomavirus.

^a^ The numbers 1 to 5 correspond to the disease state transitions represented in Figure 1.

Among patients with head and neck carcinoma, most recurrences occurred within the first 2 years after treatment.^36^ The references used for our pretraining models further demonstrate that the greatest risk is during the first year. Therefore, we divided the risk of recurrence into 3 periods: 1 year, 2 years, and 3 or more years after treatment. We enforced the risk of recurrence to either stay the same or decrease over time. This assumption was supported by the trends in the literature as well as clinical experience.

### Statistical Analysis

Published Kaplan-Meier curves were digitized using the online software WebPlotDigitizer, version 4.3 (Ankit Rohatgi). We reconstructed the individual patient data using the algorithm described by Guyot and colleagues.^37^ This method has previously been used in the development of Markov models from time–to–end point data.^17^ The algorithm was implemented using the statistical software R, version 4.02 (R Group for Statistical Computing). We then extracted transition probabilities from the reconstructed cohorts for both HPV-positive and HPV-negative cases.

#### Model Training

Our next step was to fit our HPV-adapted model to tumor stage. Training data were extracted from the NCDB. Patients with oropharyngeal cancer treated with primary radiotherapy from 2010 to 2015 were included. Surgical treatment for patients was allowed in the setting of salvage therapy. Only patients with HPV status and staging information were included (pathologic stage was used for patients treated with surgical salvage). Only patients from academic or research programs or from integrated network cancer programs were included. Patients who received palliative care and those with detectable posttreatment metastases were excluded.

Before training, we compared the NCDB data with our external validation data set: the multicenter International Collaboration on Oropharyngeal Cancer Network for Staging (ICON-S) study from 2016.^31^ Our comparison was made using a log-rank test, with a Bonferroni-corrected *P* value of .02. Our goal was to evaluate whether differences between the trained cohorts and the validation cohorts would be due to intrinsic differences between the data or to the model training process.

Within each cohort, we assumed that mortality was not dependent on pretreatment stage. Therefore, differences in cohort survival depended entirely on HPV status, whether the recurrence was local or metastatic, and the number of patients who developed recurrent disease. This constraint was due to the NCDB data being poorly annotated for recurrence timing but providing robust overall survival data.

An algorithm of the training process is represented in eFigure 2 in the Supplement. In brief, pretraining recurrence probabilities were iteratively modified by a range of multipliers. The multipliers that generated a cohort with the most similar survival outcomes as the training data were selected. The process was repeated until the training fold produced insignificant differences in the trained probabilities. eTable 1 in the Supplement contains the posttraining recurrence probabilities. Trained cohorts were subsequently compared with the NCDB cohorts using the log-rank test, with a Bonferroni-corrected α = .02.

After training, the models were compared with the ICON-S validation data. The modeled cohort size for this comparison was set at 10 000 patients (outcomes did not appreciably change with larger cohorts). In total, 6 trained cohorts (HPV positive and HPV negative, divided by disease stages III, IVA, and IVB from the American Joint Committee on Cancer, 7th edition) were compared against an analogous 6 cohorts from ICON-S. Significance was measured using the log-rank test, with Bonferroni-corrected α = .02. The model was implemented in Python, version 3.7.6 (Python Software Foundation). The Python packages used for the model are provided in eTable 4 in the Supplement.

#### Surveillance Optimization

The 6 models (HPV positive and HPV negative for disease stages III, IVA, and IVB) were each used to produce 3-year disease trajectories for 2500 simulated patients. The choice of 2500 patients empirically resulted in stable model outcomes, with larger cohorts not resulting in significant differences in the optimal regimen. The times of recurrence were used as the input for optimization.

Each generated surveillance regimen included a PET scan at month 3, consistent with the National Comprehensive Cancer Network (NCCN) version 1.2021 guidelines on oropharyngeal malignant neoplasms.^8^ A sequential grid search was performed including varying numbers of CT scans (between 1 and 6 additional scans). Latency (defined as the time between the onset of a recurrence and its discovery) was calculated for each surveillance regimen, and an optimal regimen that yielded the lowest total latency was selected. Scans were assumed to have perfect sensitivity and specificity. This assumption facilitated our optimization goal of clustering scans around time points of greater recurrence density. The upper limit of 7 total scans was chosen because it correlates to 1 scan for each NCCN-recommended clinical follow-up visit.

We compared our optimized regimens against a “standard” regimen designed by scheduling CT scans using the NCCN guidelines. We evaluated regimens using 3 metrics: sensitivity, mean latency, and number of false-positive results. We determined significant differences between the standard regimen and the optimized regimen of PET plus 6 CT scans (chosen for its equal number of scans) using an unpaired *t* test (for latency) and *z* scores for population proportions (for sensitivity and number of false-positive results). The α level was set at .008 after Bonferroni correction for 6 comparisons.

We also compared the model-informed regimens with a regimen designed using the eviCore 2.1 Clinical Guidelines for Oncology Imaging^9^ and using the same metrics as the NCCN comparison. The eviCore guidelines recommend a standard PET scan at month 3, then CT scans at month 6 and then annually (ie, months 12, 24, and 36). We compared this reimbursement-based strategy to an optimized regimen with the same number of scans (5 total).

These simulations used test characteristics for PET-CT scans, CT scans of the neck, and CT scans of the chest taken from the literature.^21,36,38,39,40,41,42^ Pooled sensitivities and specificities are provided in eFigure 3A and B in the Supplement. Computed tomography scans of the neck were able to detect only a local recurrence, whereas CT scans of the chest were able to detect only metastatic disease; PET-CT scans could detect both.

## Results

### Training Cohort Characteristics

The NCDB training data consisted of 2159 total patients (1708 men [79.1%]; median age, 59.6 years [range, 40-90 years]; 401 patients with stage III disease, 1415 patients with stage IVA disease, and 343 patients with stage IVB disease). Cohorts predominantly had HPV-negative disease (1606 [74.4%]) (Table 2). The mean (SD) follow-up was 30.5 (21.5) months. A total of 16 009 of 17 763 patients (90.1%) with OPC within the NCDB database had overall survival information available. No participants within our training cohorts were lost to follow-up (all have survival data available).

**Table 2.** Patient Summary Characteristics in the National Cancer Database Training Cohorts

Characteristic	Patients, No. (%)		
	Stage III disease (n = 401)	Stage IVA disease (n = 1415)	Stage IVB disease (n = 343)
Age, median (range), y	62 (40-90)	59 (40-90)	59 (40-90)
Sex			
Male	305 (76.1)	1131 (79.9)	272 (79.3)
Female	96 (23.9)	284 (20.1)	71 (20.7)
HPV status			
Positive	88 (21.9)	387 (27.3)	78 (22.7)
Negative	313 (78.1)	1028 (72.7)	265 (77.3)
Charlson Comorbidity Index			
0	307 (76.6)	1171 (82.8)	268 (78.1)
1	67 (16.7)	180 (12.7)	54 (15.7)
2	19 (4.7)	41 (2.9)	10 (2.9)
≥3	8 (2.0)	23 (1.6)	11 (3.2)
Grade			
Low (I or II)	184 (45.9)	517 (36.5)	127 (37.0)
High (III or IV)	128 (31.9)	405 (28.6)	92 (26.8)
Other	89 (22.2)	493 (34.8)	124 (36.2)
Chemotherapy			
Received	292 (72.8)	1214 (85.8)	308 (89.8)
None	109 (27.2)	201 (14.2)	35 (10.2)
Surgery			
Received	97 (24.2)	326 (23.0)	33 (9.6)
None	304 (75.8)	1089 (77.0)	310 (90.4)
Immunotherapy			
Received	18 (4.5)	65 (4.6)	21 (6.1)
None	383 (95.5)	1350 (95.4)	322 (93.9)

Abbreviation: HPV, human papillomavirus.

### Model Training and Validation

In the pretraining comparison of the NCDB cohorts with the ICON-S validation cohorts, survival for all disease stage–matched and HPV status–matched cohorts were statistically indistinguishable except for the cohort with stage IVA HPV-positive OPC (eFigure 4 in the Supplement). The NCDB cohort with stage IVA HPV-positive OPC demonstrated a significantly greater mortality than its ICON-S counterpart.

Trained models were then compared with their analogous NCDB training counterparts (eFigure 5A in the Supplement). There was no significant difference between any of the respective pairs (stage III HPV-positive, stage III HPV-negative, stage IVA HPV-positive, stage IVA HPV-negative, stage IVB HPV-positive, and stage IVB HPV-negative models). The comparison between the model and the ICON-S validation data is represented in eFigure 5B in the Supplement. A breakdown of the proportions of each type of recurrence can be seen in eFigure 6 in the Supplement. The external validation showed no significant difference between the stage III HPV-positive, stage III HPV-negative, stage IVA HPV-negative, stage IVB HPV-positive, and stage IVB HPV-negative models compared with their ICON-S counterparts. There were significant differences between the model and the stage IVA HPV-positive cohort, consistent with the pretraining comparison.

### Surveillance Optimization

For each cohort and number of scans, we selected the regimen that minimized mean latency. The process of optimization is depicted in eFigure 7 in the Supplement. Across all cohorts, an increase in the number of permitted scans was associated with decreased latency.

**Figure 2.**
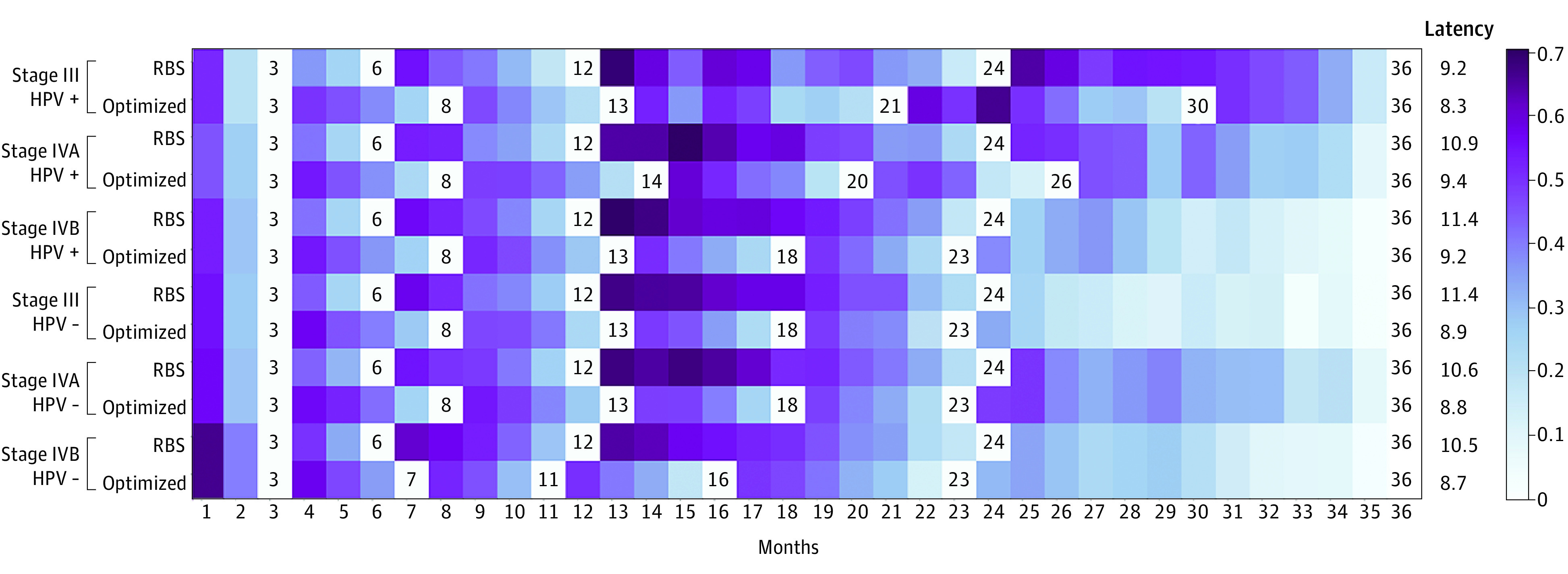
Comparison of Mean Latency Between Reimbursement-Based Schedule (RBS) and Model-Optimized Strategies The numbers on the chart refer to the scheduled month of surveillance for that regimen. The darker shades of color reflect greater mean latencies. Regimen latency was normalized using the log (1 + *z* score − [minimum *z* score of all latencies]). In-practice, latency corresponds to the data within eTable 2 in the Supplement. HPV − indicates human papillomavirus negative; HPV +, human papillomavirus positive.

Table 3 contains the performance of our optimized regimens. Compared with the standard regimen, optimized schedules of a PET scan plus 6 CT scans were associated with lower latencies (mean improvement in 0.6 months [95% CI, 0.5-0.8 months]). All differences were significant except for the cohort with stage III HPV-positive OPC. In all cases, these strategies yielded sensitivities within 0.01 of each other (no statistically significant differences). When the reimbursement-based strategy is compared with optimized regimens, the optimized regimens were associated with lower mean latencies across all cohorts (mean improvement in 1.8 months [95% CI, 1.3-2.3 months]). These differences were significant. The reimbursement-based regimen had superior sensitivities for the cohorts with stage III and IVA HPV-positive OPC, whereas the optimized regimen sensitivities were significantly better for the cohorts with stage IVB HPV-positive, stage III HPV-negative, and stage IVB HPV-negative OPC. Figure 2 shows the latency comparison between the strategies, whereas eTable 2 in the Supplement shows the complete comparison.

**Table 3.** Performance Comparison of Optimized and Standard Regimens

Regimen	Months	Sensitivity	Latency, mo	Total false-positive results per 10 000 patients
**Stage III HPV positive**				
PET scan	3	0.10	15.0	1071
Plus 1 CT scan	3, 19	0.26	12.3	2055
Plus 2 CT scans	3, 13, 28	0.41	10.6	2807
Plus 3 CT scans	3, 12, 21, 30	0.52	9.1	3624
Plus 4 CT scans	3, 8, 13, 21, 30	0.55	8.1	4110
Plus 5 CT scans	3, 8, 13, 18, 23, 30	0.56	7.8	4755
Plus 6 CT scans	3, 8, 13, 18, 23, 28, 33	0.65	7.1	5214
Standard^a^	3, 6, 9, 12, 18, 24, 36	0.64	7.4	5224
**Stage IVA HPV positive**				
PET scan	3	0.13	17.6	1014
Plus 1 CT scan	3, 18	0.31	14.3	1816
Plus 2 CT scans	3, 12, 22	0.45	11.6	2549
Plus 3 CT scans	3, 8, 14, 23	0.48	10.3	3201
Plus 4 CT scans	3, 8, 14, 20, 26	0.55	9.5	3754
Plus 5 CT scans	3, 7, 12, 17, 22, 27	0.61	8.3	4264
Plus 6 CT scans	3, 7, 11, 15, 19, 23, 31	0.67	7.7^b^	4569
Standard^a^	3, 6, 9, 12, 18, 24, 36	0.67	8.4^b^	4668
**Stage IVB HPV positive**				
PET scan	3	0.15	19.2	733
Plus 1 CT scan	3, 18	0.38	15.0	1321
Plus 2 CT scans	3, 13, 23	0.50	12.9	1732
Plus 3 CT scans	3, 8, 15, 23	0.56	11.1	2236
Plus 4 CT scans	3, 8, 13, 18, 23	0.63	9.3	2621
Plus 5 CT scans	3, 6, 9, 13, 18, 23	0.66	8.3	2977
Plus 6 CT scans	3, 6, 9, 13, 18, 23, 30	0.71	7.9^b^	3306
Standard^a^	3, 6, 9, 12, 18, 24, 36	0.71	8.4^b^	3252
**Stage III HPV negative**				
PET scan	3	0.16	19.4	798
Plus 1 CT scan	3, 18	0.39	15.2	1445
Plus 2 CT scans	3, 13, 23	0.53	12.6	1969
Plus 3 CT scans	3, 8, 15, 23	0.61	10.3	2507
Plus 4 CT scans	3, 8, 13, 18, 23	0.67	8.8	2985
Plus 5 CT scans	3, 6, 10, 14, 18, 23	0.69	7.8	3351
Plus 6 CT scans	3, 6, 9, 12, 15, 19, 23	0.72	7.0^b^	3704
Standard^a^	3, 6, 9, 12, 18, 24, 36	0.72	8.1^b^	3713
**Stage IVA HPV negative**				
PET scan	3	0.16	17.9	611
Plus 1 CT scan	3, 19	0.37	14.4	1127
Plus 2 CT scans	3, 10, 23	0.48	12.0	1602
Plus 3 CT scans	3, 9, 16, 23	0.56	10.0	2091
Plus 4 CT scans	3, 8, 13, 18, 23	0.61	8.5	2333
Plus 5 CT scans	3, 8, 13, 18, 23, 30	0.66	8.2	2705
Plus 6 CT scans	3, 6, 10, 14, 18, 23, 30	0.70	7.2^b^	2973
Standard^a^	3, 6, 9, 12, 18, 24, 36	0.69	7.9^b^	2928
**Stage IVB HPV negative**				
PET scan	3	0.23	19.1	368
Plus 1 CT scan	3, 15	0.44	14.7	643
Plus 2 CT scans	3, 11, 22	0.58	11.9	922
Plus 3 CT scans	3, 7, 13, 23	0.64	10.1	1108
Plus 4 CT scans	3, 7, 11, 16, 23	0.70	8.3	1342
Plus 5 CT scans	3, 6, 9, 13, 18, 23	0.72	7.7	1533
Plus 6 CT scans	3, 6, 9, 12, 16, 20, 24	0.75	6.9^b^	1738
Standard^a^	3, 6, 9, 12, 18, 24, 36	0.75	7.6^b^	1708

Abbreviations: CT, computed tomography; HPV, human papillomavirus; PET, positron emission tomography.

^a^ Standard refers to a PET scan at month 3 and CT scans of the neck or chest at months 6, 9, 12, 18, 24, and 36. Latency for radiologically discovered disease is defined as latency = month of radiologic disease discovery − month of recurrence onset, and latency for radiologically missed disease is defined as latency = 36 − month of recurrence onset.

^b^ Denotes when there is a significant difference between latency of PET scan plus 6 CT scans and standard regimens (unpaired *t* test; α = .008). There were no significant differences in sensitivity or false-positive results between these regimens across all cohorts (*z* score for population proportions, α = .008).

## Discussion

Our study sought to identify how posttreatment surveillance for OPC could be optimally scheduled to discover clinically silent recurrent disease. First, we developed an analytical model to simulate the complex interplay between HPV status, disease stage, local recurrence, distant recurrence, and mortality. Our model produced statistically indistinguishable survival curves for all cohorts of the ICON-S validation data set except for the cohort with stage IVA HPV-positive OPC. The high accuracy of our model in simulating patient outcomes for an external cohort supports its use for simulating patient-level recurrence data for optimal use of scans. The model results suggest that the optimal time for a scan is dependent on tumor stage and HPV status as well as the total number of allotted scans. This outcome is consistent with our expectations because both later stage of disease and HPV-negative disease are associated with increased rates of recurrence. Because our model may overestimate mortality in stage IVA HPV-positive disease, we also performed a sensitivity analysis using a modified model with adjusted recurrence rates to fit the ICON-S data (eFigure 8 in the Supplement). We found that the performance of our optimized regimen did not appreciably change when using the model with improved survival fit (eTable 3 in the Supplement).

In the comparison between our optimized regimens and the reimbursement-based strategy, our regimens produced lower sensitivities for the cohorts with stage III and IVA HPV-positive disease. We attribute this difference to our model’s freedom to create schedules that terminate prior to the end of the allotted simulation window of 36 months. In cohorts with a greater proportion of late recurrences, this model is associated with reduced regimen sensitivity.

Our study is similar to the work by Ng et al,^28^ who developed a Markov model for HNC that was used for surveillance optimization. The same group found that imaging beyond 2 years after treatment was low yield and high cost.^43^ These results are consistent with our optimization, which tended to cluster imaging studies closer to the first 2 years of follow-up. Ng et al^43^ raised questions about whether earlier detection based solely on imaging would translate into improved survival. They also noted that the ability to stratify surveillance by stage and HPV status, as our model does, could increase the value of early disease detection.

Our findings suggest that clinicians can tailor their posttreatment surveillance regimens based on patients’ disease characteristics. A 1-size-fits-all approach does not reflect the heterogenous natural history of OPC. This study also raises questions about the utility of aggressive surveillance, even in the context of a disease with high mortality. Studies by Imbimbo et al^4^ and Kim et al^36^ have found that more recurrent diseases are discovered with radiologic surveillance than with current strategies, and this finding did not translate into improved survival. Furthermore, the work by Gharzai et al^44^ addressing patient attitudes toward OPC cancer surveillance found that uniformly applied surveillance guidelines lead to an undue burden on patients with low-stage, HPV-positive disease. In their survey study, the majority of such patients preferred a less intensive surveillance strategy with fewer in-person visits. The burden of surveillance came in the form of driving distance, nonmedical costs, and time off work. Our model, which allows for less intensive surveillance strategies, offers a starting point for the development of risk-stratified surveillance schedules that could alleviate some of these challenges.

Another challenge of frequent surveillance is increased false-positive results, which are associated with unnecessary biopsies, emotional burden, and undue costs. Because most previous studies did not stratify their surveillance regimens based on tumor characteristics, as does our model, the effectiveness of our model-generated regimens is still unknown. Overall, clinicians should remain simultaneously aware of both the patterns of OPC recurrence and the morbidity associated with testing errors without providing gains in mortality.

### Limitations

Our work has several limitations. First, while the studies used to build our pretraining models were stratified by HPV status, they often did not control for several factors that have known associations with survival and recurrence, such as age, tobacco and alcohol use, and specific tumor and nodal stage (as opposed to overall disease stage). As such, it is possible that the designation of HPV-positive vs HPV-negative cohorts contains risk-related information beyond HPV status.

Another limitation is that our training cohorts consisted of a majority of HPV-negative patients, whereas HPV-related cancers have significantly increased in prevalence. As such, the generalizability of the findings may be limited.

Our model also assumes that the differences in survival between the different stages of disease are associated entirely with the rate of recurrence and, as a corollary, treats all recurrent diseases of a given HPV status, once they have recurred, the same. This assumption does not coincide perfectly with tumor biology but was necessary for stage stratification. Finally, our model assumes perfect patient adherence to follow-up, whereas in 1 study, as many as 20% of patients with HNC were not very adherent to follow-up visits.^45^ Prospective, practice-based clinical studies are essential in determining whether our individualized approach to surveillance in fact produces improved outcomes.

## Conclusions

Our study has demonstrated how optimal surveillance regimens for OPC can differ based on tumor stage and HPV status. It also demonstrates that the incorporation of additional posttreatment imaging was associated with diminishing returns. These simulations are a valuable tool in developing more standardized guidelines on posttreatment surveillance. Future efforts in determining the cost-effectiveness of optimized surveillance regimens are a natural extension of our work. Furthermore, the techniques used in this study are not limited to OPC but rather can be generalized to other cancer types and risk factors in the hope of generating more effective, patient-personalized surveillance.

## Notes

Supplement. **eTable 1.** Posttraining Transition Probabilities Stratified by Stage and HPV Status **eTable 2.** Performance of Reimbursement-Based Schedule (RBS) vs Optimized Regimens **eTable 3.** Modified Stage IVA HPV-Positive Cohort Performance **eTable 4.** Python Packages Used in Model Development **eFigure 1.** Flowchart of Model Training **eFigure 2.** Recurrence Model Training Algorithm **eFigure 3A.** Test Characteristics of PET-CT Scan for Recurrent Head and Neck Disease **eFigure 3B.** Test Characteristics of CT Scan for Recurrent Head and Neck Disease **eFigure 4.** Comparison Between Training Cohort (NCDB) and External Validation Cohort (ICON-S) **eFigure 5.** Cohort-Specific Comparison of Overall Survival **eFigure 6.** Disease-Free Survival Stratified by Stage and HPV Status and Disease State (Locoregional and Distant Metastasis) **eFigure 7.** Model-Based Recommendations Adapt to Minimize Latency Based on Overall Number of Scans **eFigure 8.** Modified Stage IVA HPV-Positive Cohort Compared With ICON-S Counterpart
