# Supplementary material for: Development and Validation of a Decision Analytical Model for Posttreatment Surveillance for Patients With Oropharyngeal Carcinoma
Source: JAMA Netw Open. 2022 Apr 13;5(4):e227240. doi: 10.1001/jamanetworkopen.2022.7240 (PMC9008506; doi:10.1001/jamanetworkopen.2022.7240)
Supplement: Supplement. — eTable 1. Posttraining Transition Probabilities Stratified by Stage and HPV Status eTable 2. Performance of Reimbursement-Based Schedule (RBS) vs Optimized Regimens eTable 3. Modified Stage IVA HPV-Positive Cohort Performance eTable 4. Python Packages Used in Model Development eFigure 1. Flowchart of Model Training eFigure 2. Recurrence Model Training Algorithm eFigure 3A. Test Characteristics of PET-CT Scan for Recurrent Head and Neck Disease eFigure 3B. Test Characteristics of CT Scan for Recurrent Head and Neck Disease eFigure 4. Comparison Between Training Cohort (NCDB) and External Validation Cohort (ICON-S) eFigure 5. Cohort-Specific Comparison of Overall Survival eFigure 6. Disease-Free Survival Stratified by Stage and HPV Status and Disease State (Locoregional and Distant Metastasis) eFigure 7. Model-Based Recommendations Adapt to Minimize Latency Based on Overall Number of Scans eFigure 8. Modified Stage IVA HPV-Positive Cohort Compared With ICON-S Counterpart [file jamanetwopen-e227240-s001.pdf]

Supplementary Online Content

Nair V, Auger S, Kochanny S, et al. Development and validation of a decision analytical model for posttreatment surveillance for patients with oropharyngeal carcinoma. *JAMA Netw Open.* 2022;5(4):e227240. doi:10.1001/jamanetworkopen.2022.7240

eTable 1. Posttraining Transition Probabilities Stratified by Stage and HPV Status
eTable 2. Performance of Reimbursement-Based Schedule (RBS) vs Optimized Regimens
eTable 3. Modified Stage IVA HPV-Positive Cohort Performance
eTable 4. Python Packages Used in Model Development
eFigure 1. Flowchart of Model Training
eFigure 2. Recurrence Model Training Algorithm
eFigure 3A. Test Characteristics of PET-CT Scan for Recurrent Head and Neck Disease
eFigure 3B. Test Characteristics of CT Scan for Recurrent Head and Neck Disease
eFigure 4. Comparison Between Training Cohort (NCDB) and External Validation Cohort (ICON-S)
eFigure 5. Cohort-Specific Comparison of Overall Survival
eFigure 6. Disease-Free Survival Stratified by Stage and HPV Status and Disease State (Locoregional and Distant Metastasis)
eFigure 7. Model-Based Recommendations Adapt to Minimize Latency Based on Overall Number of Scans
eFigure 8. Modified Stage IVA HPV-Positive Cohort Compared With ICON-S Counterpart

This supplementary material has been provided by the authors to give readers additional information about their work.

© 2022 Nair V et al. *JAMA Network Open.*

eTable 1. Posttraining Transition Probabilities Stratified by Stage and HPV Status

Stage	HPV-status	Year-1		Year-2		Year-3+	
		P(LR)	P(mets)	P(LR)	P(mets)	P(LR)	P(mets)
III	+	.093%	.13%	.093%	.13%	.093%	.13%
	-	1.3%	.38%	1.3%	.38%	.17%	.013%
IVA	+	.29%	.41%	.29%	.41%	.012%	.17%
	-	1.7%	.51%	1.7%	.51%	.73%	.16%
IVB	+	.88%	1.2%	.88%	1.2%	.18%	.24%
	-	4.1%	1.2%	2.9%	.88%	.84%	.062%

P(LR): per month probability of locoregional recurrence

P(mets): per month probability of metastatic disease

© 2022 Nair V et al. *JAMA Network Open.*

eTable 2. Performance of Reimbursement-Based Schedule (RBS) vs Optimized Regimens

cohort	RBS			optimized		
	Sensitivity	Mean Latency	False Positives	Sensitivity	Mean Latency	False Positives
III HPV+	.58*	9.2‡	4223	.52*	8.3‡	4277
IVA HPV+	.59*	10.9‡	3673	.57*	9.4‡	3714
IVB HPV+	.61*	11.4‡	2623	.63*	9.2‡	2579
III HPV-	.61*	11.4‡	3012	.67*	8.9‡	2999
IVA HPV-	.61	10.6‡	2398	.61	8.8‡	2457
IVB HPV-	.66*	10.5‡	1343	.69*	8.7‡	1394

* Denotes sensitivities that are significantly different (z score for population proportions, alpha=0.0083)

‡ Denotes latencies that are significantly different (unpaired t-test, alpha=0.0083).

Number of false positives was not significantly different across all cohorts (z score for population proportions, alpha=0.0083)

© 2022 Nair V et al. *JAMA Network Open.*

eTable 3. Modified Stage IVA HPV-Positive Cohort Performance

Regimen	Months	Sensitivity	Latency (months)	Total false positives per 10,000 patients
PET	3	.12	18.2	1111
+1 CT	3,18	.31	14.6	1936
+2 CT	3,12,22	.44	12.1	2770
+3 CT	3,8,14,23	.50	10.3	3443
+4 CT	3,8,14,20,26	.58	9.4	4035
+5 CT	3,7,12,17,22,27	.62	8.2	4519
+6 CT	3,7,11,15,19,23,31	.67	7.7*	4935
Standard	3,6,9,12,18,24,36	.68	8.5*	5094

Standard refers to a PET at month 3, CT neck/chest at 6,9,12,18,24,36. Latency for radiologically discovered disease is defined: latency=month of radiologic disease discovery – month of recurrence onset; Latency for radiologically missed disease is defined: latency= 36 – month of recurrence onset

* Denotes when there is a significant difference between latency of PET+6CT and Standard regimens (unpaired t-test, alpha=0.0083). There were no significant differences in sensitivity or false positives between these regimens across all cohorts (z score for population proportions, alpha=0.0083).

© 2022 Nair V et al. *JAMA Network Open.*

**eTable 4.** Python Packages Used in Model Development	
Package Name	Purpose
numpy	Array data structure implementation
pandas	Data input and output; Data manipulation
scikit-learn	Model training and testing
seaborn	Data visualization

© 2022 Nair V et al. *JAMA Network Open.*

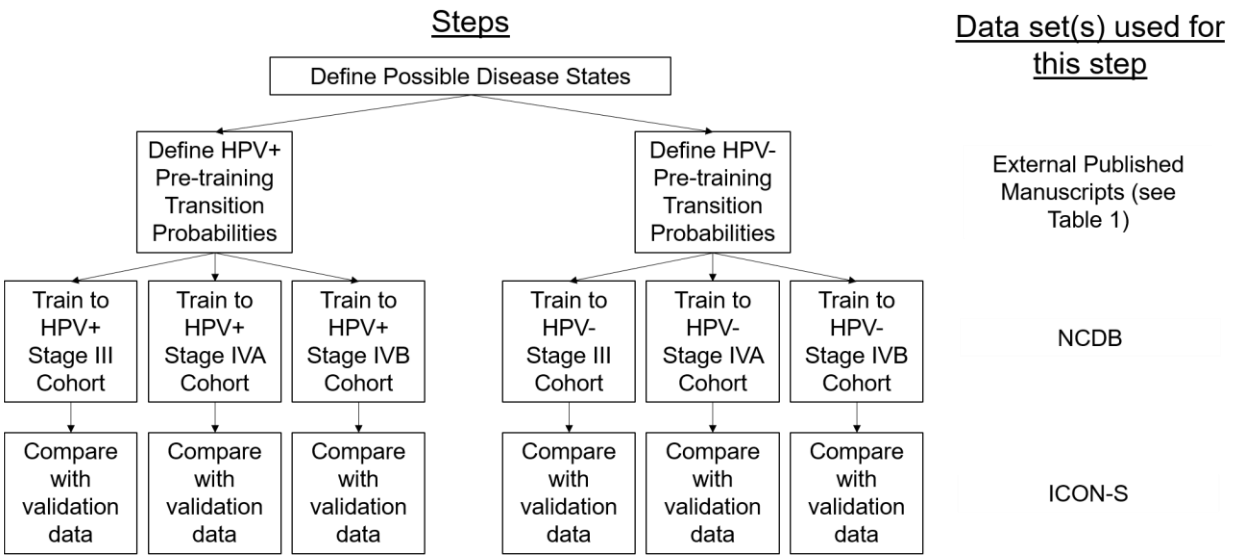

eFigure 1. Flowchart of Model Training

NCDB=National Cancer Database; ICON-S refers to the 2016 International Collaboration on Oropharyngeal cancer Network for Staging manuscript by O'Sullivan et al[27].

© 2022 Nair V et al. *JAMA Network Open.*

1. Define x time intervals (in months) for training, $t_1, t_2 \ldots t_x$
2. Define possible patient disease states (in this case: 'No disease', 'Locoregional Recurrence', 'Metastatic Disease', 'Death').
3. For each time interval, define pre-training transition probabilities P(no disease), P(LR), P(mets), P(death), in which P(LR) is the per month probability of locoregional recurrence, P(mets) is the per month probability of metastatic disease, P(death) is the per month probability of death from causes other than cancer, and P(no disease)=1-(P(LR)+P(mets)+P(death)).
4. For each time interval, P(recurrence)=P(LR)+P(mets).
5. For each time interval, define the probabilities of death from recurrent disease P(LR->death) and P(mets->death), in which P(LR->death) refers to the monthly probability of death from a locoregional recurrence and P(mets->death) refers to the monthly probability of death from metastatic recurrence.
6. Create list of multipliers, M of size k in which M=[0.1, 0.2…0.1*k].
7. For each interval, create a list of modified recurrence probabilities, Ps_mod, in which Ps_mod=M*P(recurrence) for that time interval. For each modified recurrence probability P_mod_i in Ps_mod, $P\_mod_i = M_i$*P(recurrence)=M_i*P(LR)+M_i*P(mets)
8. For each P_mod in Ps_mod, generate a cohort c of Markov chain of p patients in which each patient starts in a state of 'No disease'. Each patient is simulated for a duration of m months.
9. For patients in the cohort who achieve a state of 'Locoregional Recurrence' or 'Metastatic Disease', truncate their Markov chain up until the month of their recurrence. Then, further simulate the chain until you create a chain of m total months, looking to see if patient transitions to a state of 'Death'. Use P(LR->death) for that time interval if patient is in a state of 'Locoregional Recurrence' and use P(mets->death) for that time interval if patient is in a state of 'Metastatic Disease). You now have k total cohorts of simulated patients ($c_1, c_2 \ldots c_k$).
10. Define training cohort, $c_{training}$, which is a cohort of n patients of with pre-determined risk factors (e.g. tumor stage, tumor HPV-status).
11. Define speed of death, s, in which for a given time interval s=(number at risk at end of interval – number at risk at beginning of interval)/number of months in that time interval.
12. For $c_{training}$, create a list $s_{training}$ of s for each of the x time intervals in which $s_{training}=[s_{training1}, s_{training2} \ldots s_{trainingx}]$
13. For each cohort c_1 to c_k, create $s\_cohort_{testing}$ in which $s\_cohort_{testing}$ consists of the speed of death s for each time interval of that cohort,
14. Define difference between speeds of death, $diff$, in which $diff = (|s_{testing} - s_{training}| / s_{training})$
15. For each time interval and for each multiplier from M_1 up to M_k calculate $diff$.
16. For each time interval, select the multiplier that produces the smallest $diff$ within that time interval and | generate a new list of multipliers $M_{retrain}$ (example output of one fold of training in a model of three time intervals: $M_{retrain}$=[0.8,1.1,0.7]).
17. For each time interval j out of the k total time intervals, redefine P(LR)=P(LR)*$M_{retrain\_j}$ and P(mets)=P(mets)*$M_{retrain\_j}$.
18. Repeat steps 6 to 17 until $M_{retrain\_1}$ produces a value between 0.9 and 1.1 for two consecutive iterations (representing a series of minimally impactful training iterations indicative of relatively stable final transition probabilities).
19. Output of this training will be a set of transition probabilities in which, for each time interval, P(LR) and P(mets) have been modified in order to fit the observed survival outcomes of $c_{training}$. P(no disease) is accordingly redefined as in step (3). No other probabilities are changed.

eFigure 2. Recurrence Model Training Algorithm

© 2022 Nair V et al. *JAMA Network Open.*

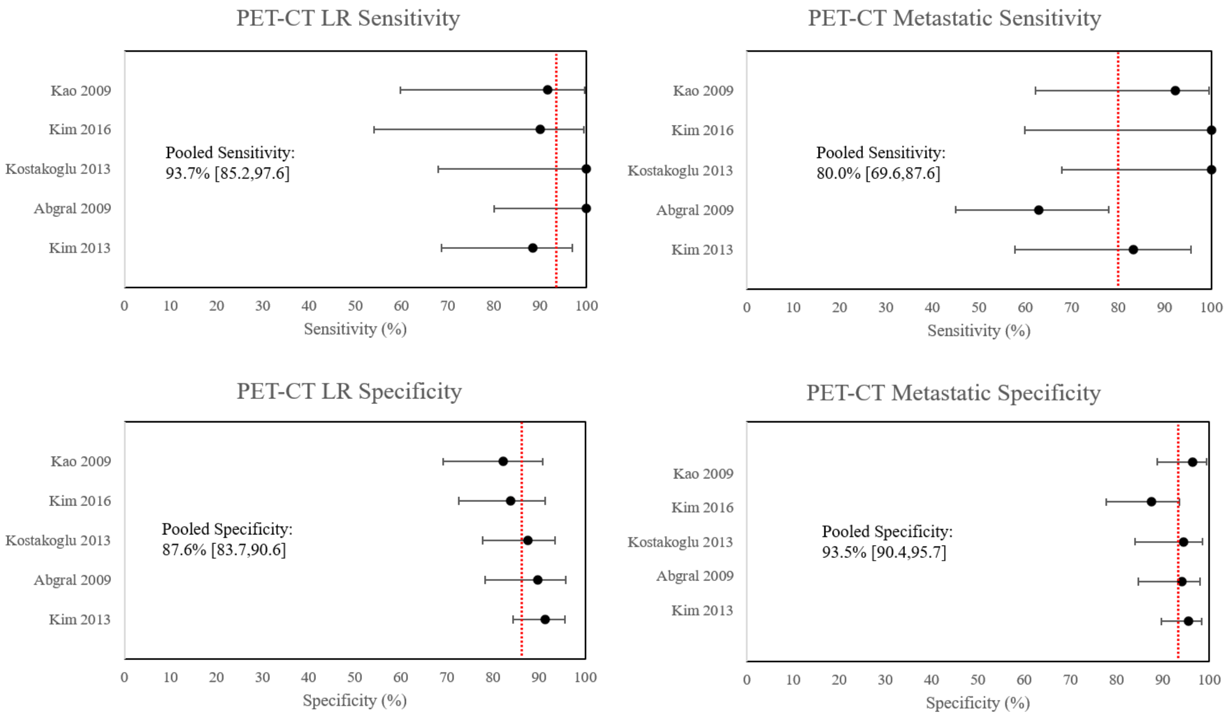

eFigure 3A. Test Characteristics of PET-CT Scan for Recurrent Head and Neck Disease

LR=locoregional recurrence, red line=pooled mean value. Test characteristics given in the form of pooled mean[95% confidence interval]

© 2022 Nair V et al. *JAMA Network Open.*

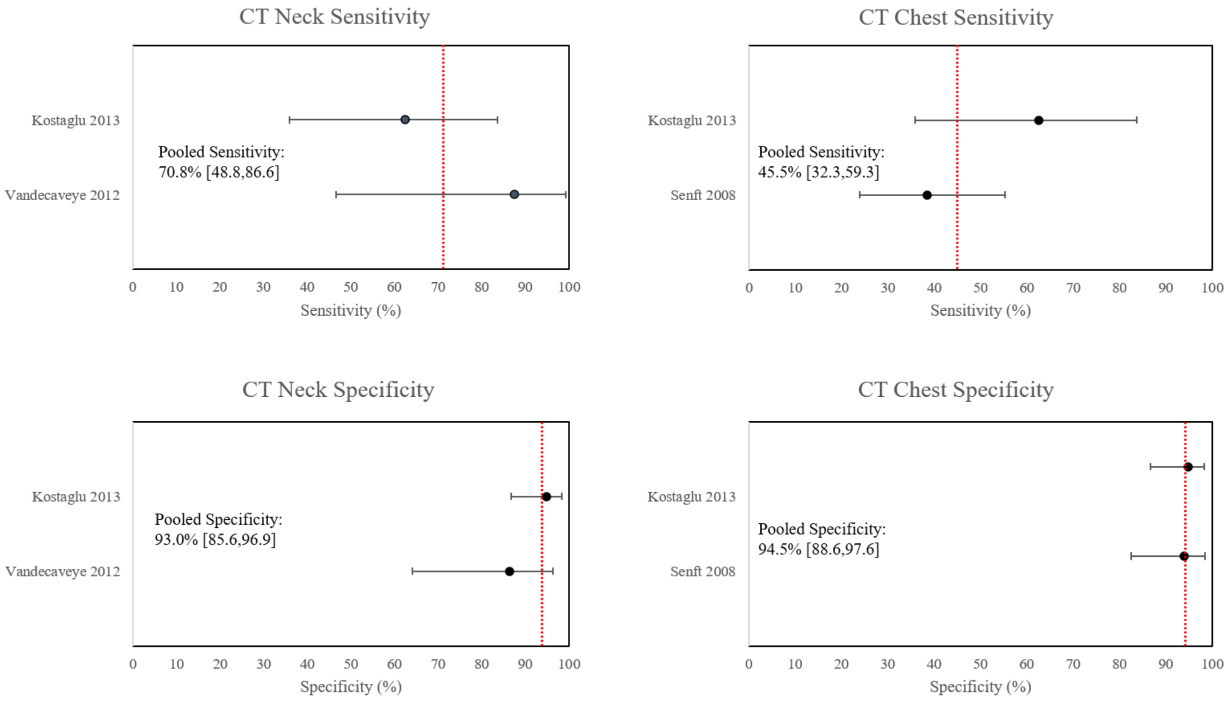

eFigure 3B. Test Characteristics of CT Scan for Recurrent Head and Neck Disease

CT Neck test characteristics were used in the simulation for detection of locoregional recurrence. CT Chest test characteristics were used in the simulation for detection of metastatic recurrence. Red line=pooled mean value. Test characteristics given in the form of pooled mean[95% confidence interval]

© 2022 Nair V et al. *JAMA Network Open.*

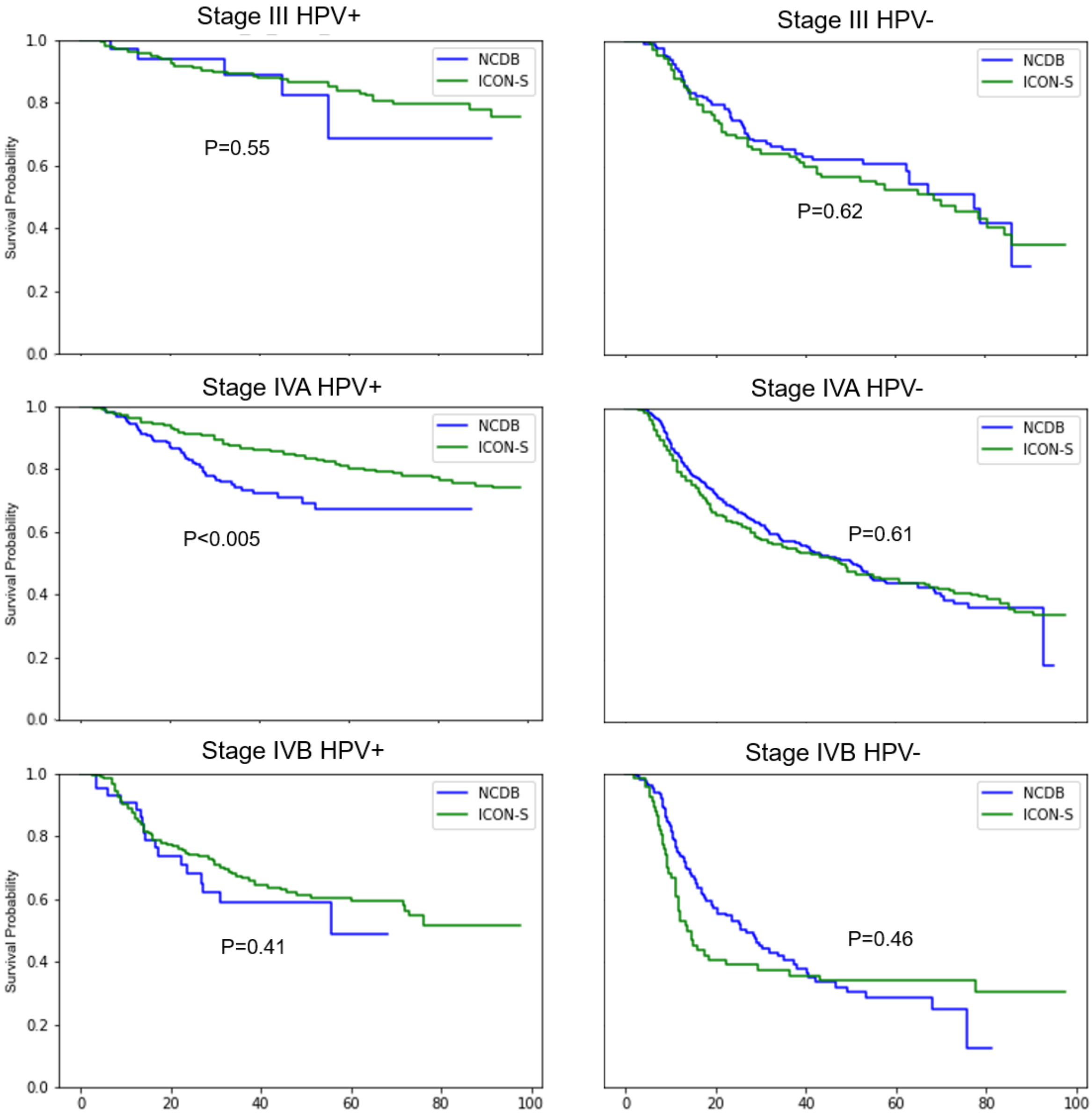

eFigure 4. Comparison Between Training Cohort (NCDB) and External Validation Cohort (ICON-S)

All p-values refer to log-rank test between cohorts of interest.

© 2022 Nair V et al. *JAMA Network Open.*

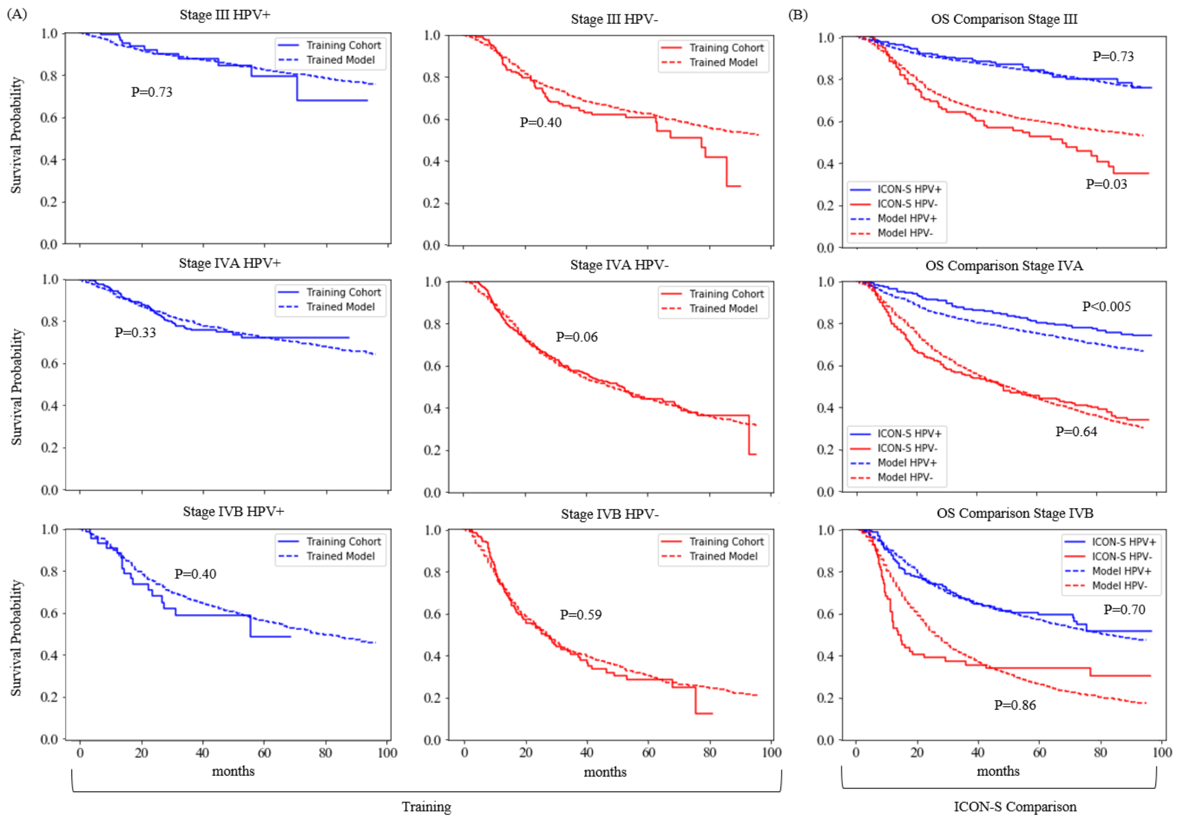

eFigure 5. Cohort-Specific Comparison of Overall Survival

(A) Comparison of the modified base model (trained model) and the training NCDB cohort. (B) External validation of trained model using ICON-S cohorts. Comparison using log-rank test.

© 2022 Nair V et al. *JAMA Network Open.*

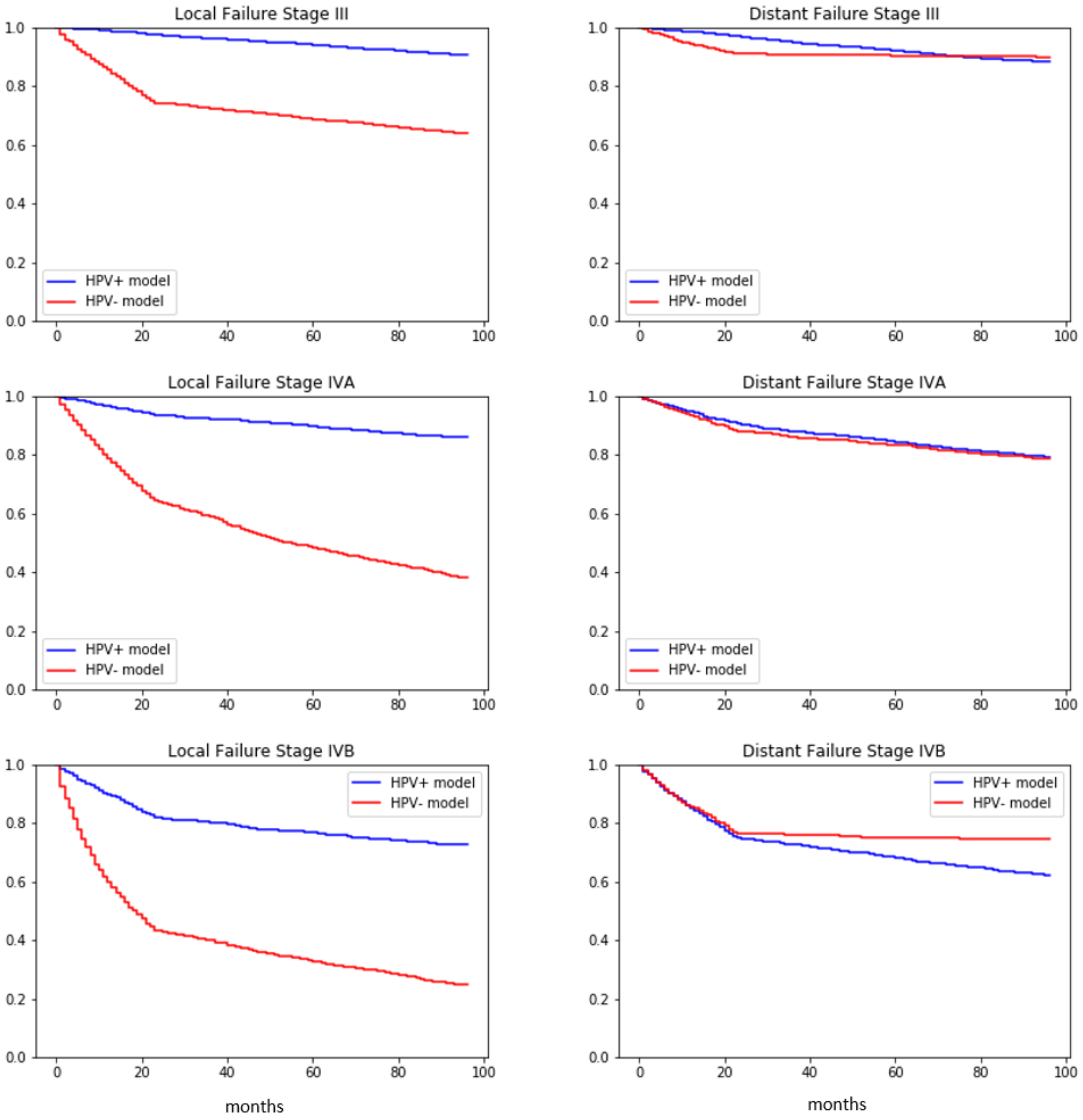

eFigure 6. Disease-Free Survival Stratified by Stage and HPV Status and Disease State (Locoregional and Distant Metastasis)

© 2022 Nair V et al. *JAMA Network Open.*

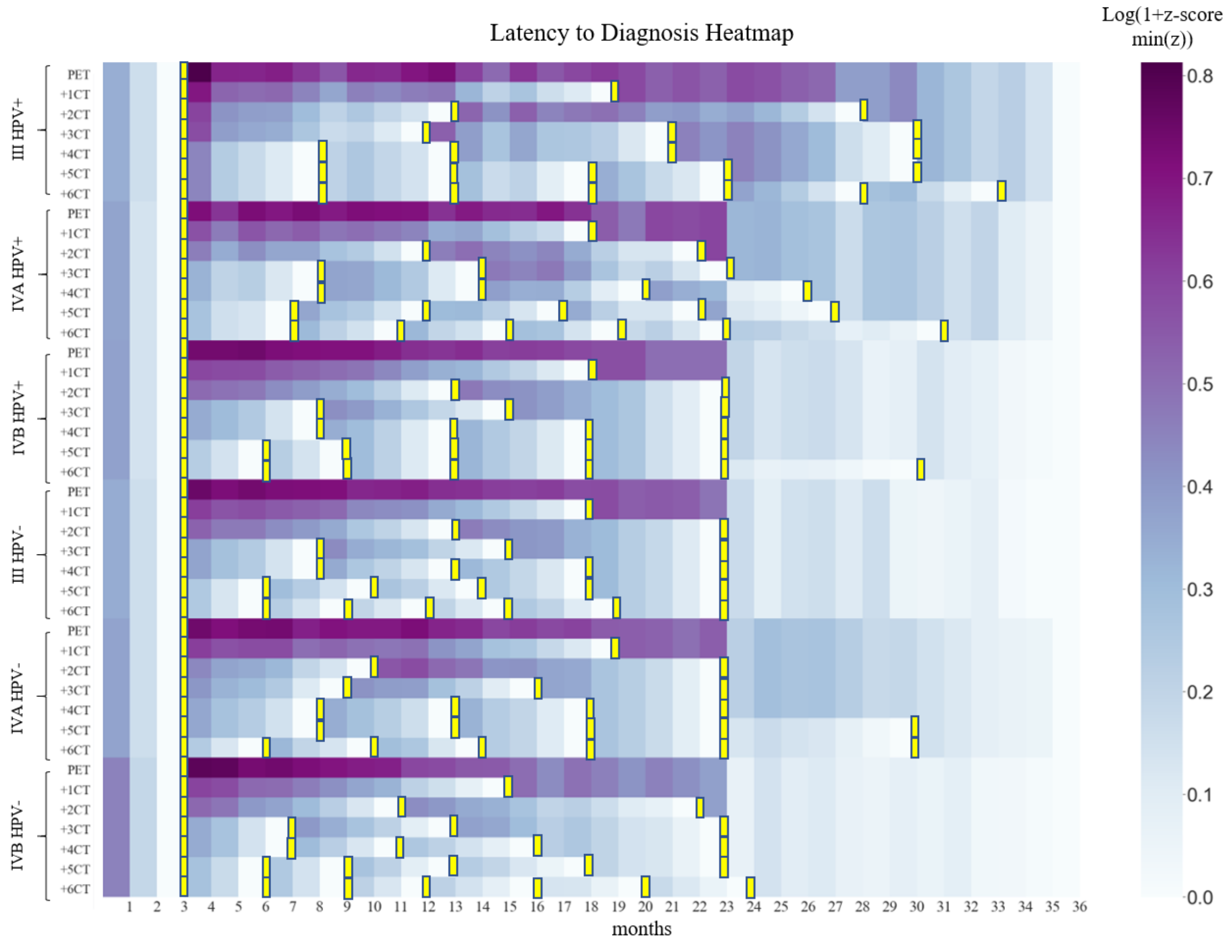

eFigure 7. Model-Based Recommendations Adapt to Minimize Latency Based on Overall Number of Scans

Regimen latency was normalized using the log(1+z-score- (minimum z-score of all latencies)). Darker colors correspond to greater latency. Each block represents one month. Each yellow bar represents the timing of a single scan.

PET=regimen with only a single PET-scan at month 3; +1CT, +2CT etc. indicates how many additional scans were allowed on top of the PET at month 3

© 2022 Nair V et al. *JAMA Network Open.*

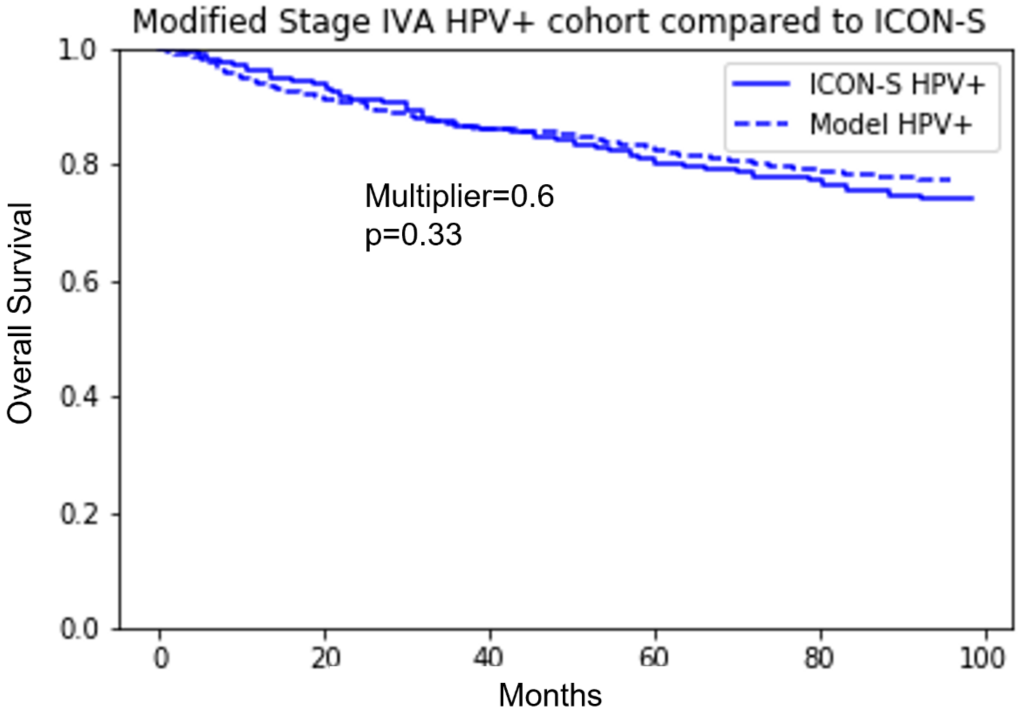

eFigure 8. Modified Stage IVA HPV-Positive Cohort Compared With ICON-S Counterpart

© 2022 Nair V et al. *JAMA Network Open.*
